# Assembly and maintenance of GABAergic and Glycinergic circuits in the mammalian nervous system

**DOI:** 10.1186/s13064-018-0109-6

**Published:** 2018-06-07

**Authors:** Clare R. Gamlin, Wan-Qing Yu, Rachel O. L. Wong, Mrinalini Hoon

**Affiliations:** 1 Department of Biological Structure, University of Washington, Seattle, WA USA; 2 Department of Ophthalmology and Visual Sciences, University of Wisconsin, Madison, WI USA

**Keywords:** Inhibition, Synaptic targeting, Synapse formation, Circuit refinement, Synapse maturation

## Abstract

Inhibition in the central nervous systems (CNS) is mediated by two neurotransmitters: gamma-aminobutyric acid (GABA) and glycine. Inhibitory synapses are generally GABAergic or glycinergic, although there are synapses that co-release both neurotransmitter types. Compared to excitatory circuits, much less is known about the cellular and molecular mechanisms that regulate synaptic partner selection and wiring patterns of inhibitory circuits. Recent work, however, has begun to fill this gap in knowledge, providing deeper insight into whether GABAergic and glycinergic circuit assembly and maintenance rely on common or distinct mechanisms. Here we summarize and contrast the developmental mechanisms that regulate the selection of synaptic partners, and that promote the formation, refinement, maturation and maintenance of GABAergic and glycinergic synapses and their respective wiring patterns. We highlight how some parts of the CNS demonstrate developmental changes in the type of inhibitory transmitter or receptor composition at their inhibitory synapses. We also consider how perturbation of the development or maintenance of one type of inhibitory connection affects other inhibitory synapse types in the same circuit. Mechanistic insight into the development and maintenance of GABAergic and glycinergic inputs, and inputs that co-release both these neurotransmitters could help formulate comprehensive therapeutic strategies for treating disorders of synaptic inhibition.

## Background: GABAergic and Glycinergic circuits in the central nervous system

**Fig. 1:**
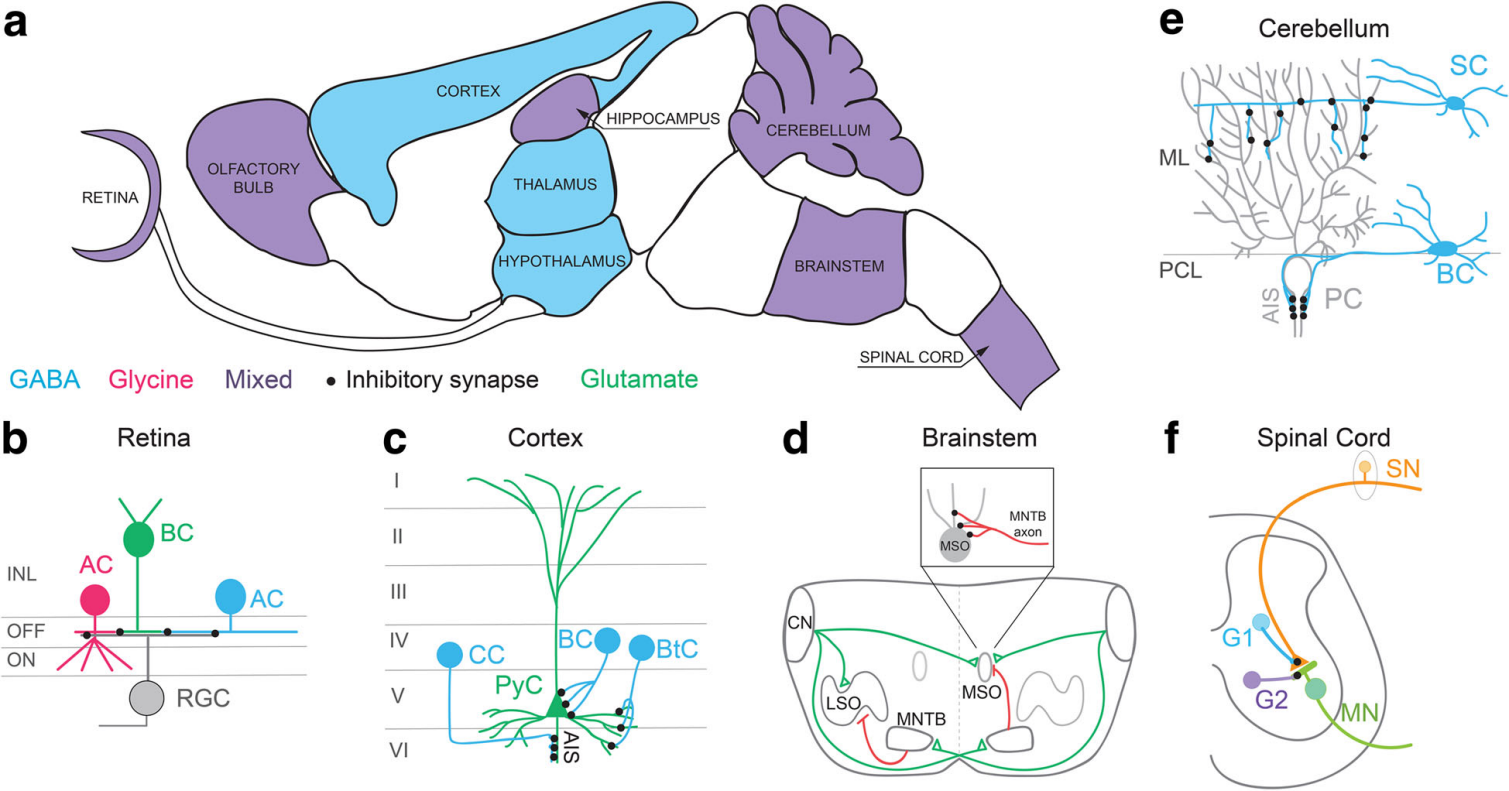
Types of inhibitory circuits across the CNS. **a** Modulation of neuronal activity in many CNS regions relies primarily on GABAergic inhibition (blue regions), whereas other regions engage both GABAergic and glycinergic inhibition (purple regions; mixed). In regions such as the retina, GABA and glycine are often released by separate populations of inhibitory neurons. However, inhibitory neurons in the spinal cord can co-release both transmitter types. Sagittal outline of the mouse brain adapted from the GENSAT brain atlas [153]. **b-f** Schematics showing outline of inhibitory circuits discussed in the review. Some circuits show laminar organization (**b**, **c**, **e**), and interneurons typically target specific subcellular compartments of their postsynaptic partners (**b**-**f**). **b** Schematic of the retina showing glycinergic and GABAergic amacrine cells (AC) contacting glutamatergic bipolar cells (BC) and retinal ganglion cells (RGC) in OFF and ON subdivisions of the inner nuclear layer (INL). [13–15]. (**c**) In the primary cortex, multiple types of GABAergic interneurons (blue) synapse onto glutamatergic pyramidal cells (PyC, green), each interneuron targeting a specific subcellular location on the PyC. For example, chandelier cells (CC) form synapses onto PyC axon initial segments (AIS). Cortical basket cells (BC) and bitufted cells (BtC) form synapses onto the soma and distal dendrites of PyCs, respectively. Summarized from: [27, 141]. (**d**) Schematic of circuits between mammalian brainstem nuclei. Dotted grey line represents the midline of the cross-section through the brainstem. The lateral superior olive (LSO) neurons receive excitatory input from the ipsilateral cochlear nucleus (CN) and inhibitory glycinergic input from the ipsilateral medial nucleus of the trapezoid body (MNTB), which is driven by the contralateral CN. Medial superior olive (MSO) neurons receive excitatory input from both ipsi- and contralateral CN, as well as inhibitory glycinergic input from the ipsilateral MNTB. MNTB axons provide glycinergic inhibition onto the soma of MSO neurons. Summarized from [29]. (**e**) Cerebellar inhibitory circuits. In the cerebellum, GABAergic stellate cells (SC) and basket cells (BC) target distinct subcellular compartments of Purkinje cells (PC). Summarized from [33, 36]. ML: Molecular layer, PCL: Purkinje cell layer, AIS: Axon initial segment. (**f**) Schematic of a spinal cord inhibitory circuit. Distinct inhibitory interneurons (G1 and G2), which are GABAergic and/or mixed GABA/glycinergic, target sensory afferents (SN) and motor neurons (MN) in the spinal cord, respectively. Summarized from [21, 22]

Signal processing in neural circuits relies on a balance between excitation and inhibition. Inhibition not only truncates action potential firing of principal neurons, it is also involved in expanding the computational power and feature selectivity of a circuit [1]. There are two major inhibitory neurotransmitters that modulate excitatory signals in the CNS: γ-aminobutyric acid (GABA) and glycine. Inhibitory circuits across different brain regions rely preferentially on GABAergic or glycinergic transmission, but some neural circuits utilize both GABA and glycine at an individual synapse [2]. Immunohistochemical and electrophysiological techniques have helped define the distribution of GABAergic and glycinergic circuits in the CNS (examples shown in Fig. 1A). Brain regions such as the cortex, hypothalamus and lateral geniculate nucleus (LGN) within the thalamus primarily use GABAergic interneurons for signal modulation (reviewed by [3–5]). Regions such as the retina, spinal cord, brainstem nuclei, cerebellum, olfactory bulb and hippocampus, however, engage both GABAergic and glycinergic inhibition [2, 6–10]. In regions such as the retina, GABAergic and glycinergic inhibition can act separately or together to modulate signal processing and shape output [11]. GABA and glycine can also be co-released from the axon terminal of an individual interneuron allowing a wider dynamic range of inhibitory modulation than could be conferred by the action of a single neurotransmitter type [12].

In this review, we highlight commonalities and differences in the cellular and molecular mechanisms that guide the development, maturation and maintenance of GABAergic and glycinergic circuits. We will contrast activity-dependent and independent cues that influence synaptic partner selection, synapse formation, maturation, refinement and maintenance of GABAergic and glycinergic connections in the CNS. Further, we will highlight that some GABAergic and glycinergic synapses can rely on the same synapse organizing molecules, but often the organization and maintenance of GABAergic and glycinergic circuits are regulated by distinct molecular and activity-dependent mechanisms. We provide schematics of the major inhibitory circuits that we refer to throughout this review in Fig. 1b-f.

## Synaptic partner selection

As in excitatory circuits, the first step in the assembly of inhibitory circuits is the selection of appropriate pre- and postsynaptic partners. The axonal and dendritic arbors of some inhibitory neurons, such as those in the neocortex and hippocampus, are rather elaborate, but they only connect with specific partner types. This suggests that there must exist cell-cell recognition cues that facilitate synaptic partner selection amidst a plethora of possibilities. Here, we briefly review what is known concerning the cellular and molecular cues that are involved in specifying synaptic partners of inhibitory neurons.

**Fig. 2:**
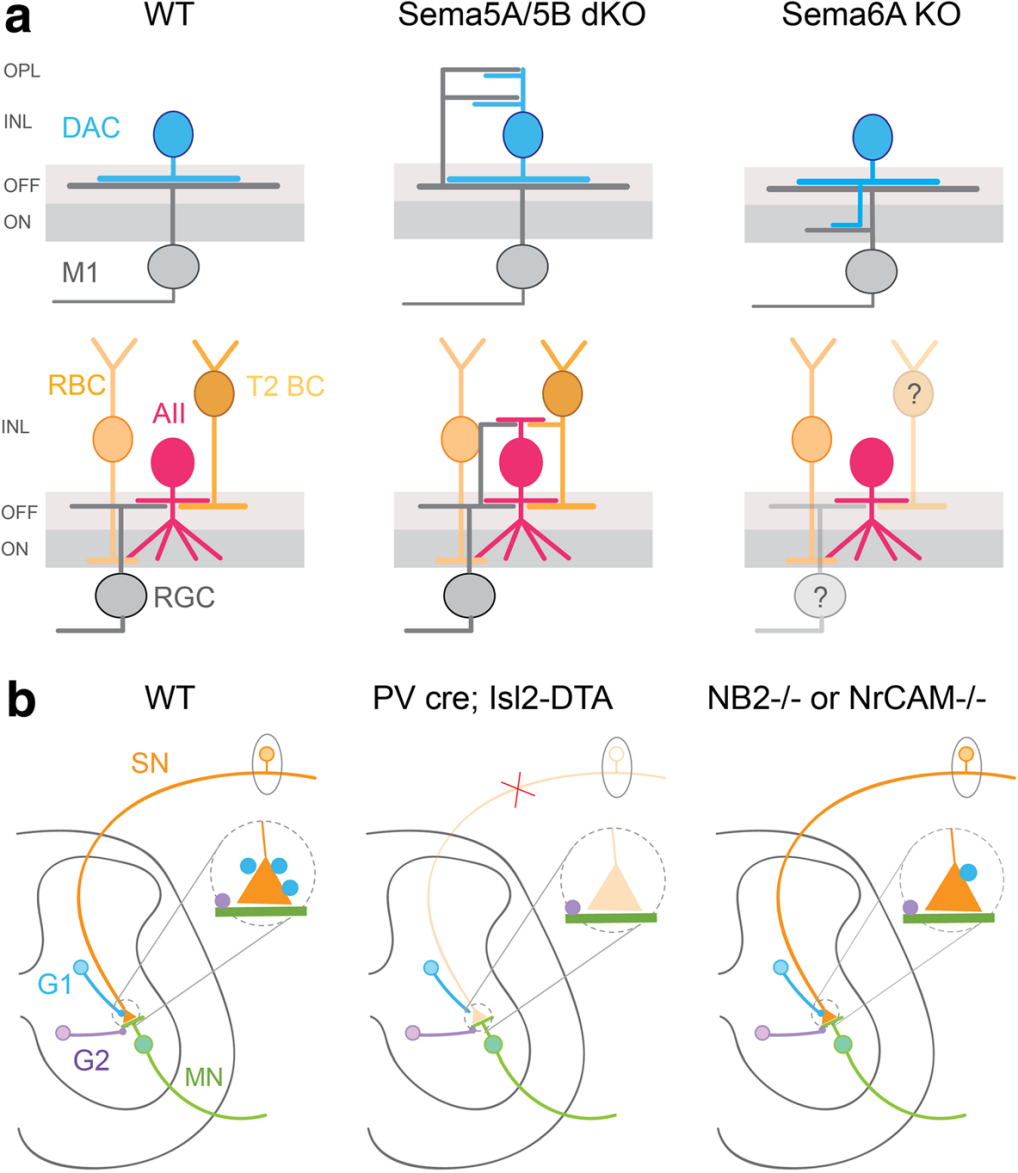
Molecular cues guide partner selection of inhibitory neurons. **a** Schematic showing the lamination of GABAergic-dopaminergic amacrine cells (DACs) and glycinergic AII amacrine cells together with their synaptic partners in wildtype (WT), Sema5A/6A double knockout mutants (dKO) and Sema6A knockouts (KO). T2 BC: Type 2 bipolar cell, M1: melanopsin-expressing retinal ganglion cell, RBC: rod bipolar cell, RGC: retinal ganglion cell, ON: inner sublamina of the retinal plexiform layer, OFF: outer sublamina of the retinal plexiform layer, INL: inner nuclear layer, OPL: outer plexiform layer. Summarized from [18, 19]. Question mark indicates non-examined synaptic partners. **b** Organization of inhibitory connections in the spinal cord sensory-motor circuit. Distinct populations of inhibitory neurons (G1 and G2) target sensory afferent terminals (SN) and motor neurons (MN), respectively, in WT mice. When sensory afferents are eliminated in PV cre/Isl2-DTA mice, G1 neurons do not form aberrant connections with motor neurons. Inhibitory synapses from G2 to motor neurons are still present in these mutants. In NB2−/− or NrCAM−/− mice, the number of inhibitory synapses from G1 to sensory neurons is significantly reduced but G2 interneuronal contacts onto motor neurons remain unaffected. G1: GABAergic neurons; G2: GABAergic and/or glycinergic neurons. Summarized from [21, 22]

The axonal and dendritic arbors of some types of inhibitory neurons are confined to specific regions of the neuropil, where they contact the processes of potential partners that also direct their arbors to these locations. Spatial restrictions in the axonal and dendritic arborizations of inhibitory neurons are particularly evident for amacrine cell interneurons of the vertebrate retina (for review on retinal organization see [13–15]). Amacrine cells are either GABAergic or glycinergic, with neurites that both provide and receive synaptic input. The processes of many types of GABAergic and glycinergic amacrine cells stratify in one or more sublaminae of the inner plexiform layer, the inner synaptic neuropil of the retina. Because the pre- and postsynaptic partners of amacrine cells also confine their axons and dendrites to specific sublaminae, amacrine cells that have stratified arbors can only contact partners whose processes costratify in the same sublaminae. Several cell adhesion molecules that regulate neurite lamination of amacrine cells have been identified. Notably, Dscam/DscamL and Sidekicks (sdk1 and sdk2) guide the specific lamination of amacrine cells and ganglion cells in developing chick retina [16, 17]. In the mouse retina, transmembrane semaphorins Sema5A and Sema5B restrict lamination of the processes of many retinal cell types in the inner plexiform layer, including both GABAergic (e.g. dopaminergic amacrine cells, DACs) and glycinergic amacrine cells (e.g. AII amacrine cells) [18]. In Sema5A/Sema5B double mutants, aberrant processes of DACs and AII amacrine cells can be observed in the inner nuclear layer and the outer plexiform layer of the retina (Fig. 2a). Within the inner plexiform layer, heterophilic repulsive interactions mediated by the guidance molecule Sema6A and its receptor, PlexinA4, have been shown to further confine lamination of amacrine cells to specific sublaminae. As such, in both the Sema6A and PlexinA4 knockout animals, aberrant processes of DACs traverse across several sub-laminae of the inner plexiform layer [19] (Fig. 2a). Although lamination of DACs is perturbed in both PlexinA4 and Sema6A mutant mice, dendritic lamination of their postsynaptic partners, the M1 ganglion cells, is equally disrupted such that DACs and M1 ganglion cells still co-laminate even in abnormal locations [19] (Fig. 2a). These observations suggest that there exist specific cell-cell recognition cues that operate independently of cues that direct neurite lamination.

Findings in the spinal cord also underscore the fact that cell-cell recognition cues enable inhibitory neurons to recognize synaptic partners locally. In the spinal cord sensory-motor circuit, distinct populations of proprioceptive sensory afferents target specific motor neurons and different populations of inhibitory neurons form synapses onto the sensory afferent terminals and motor neurons, respectively. Inhibitory synapses onto the sensory afferents are usually GABAergic, whereas those on motor neurons are GABAergic and/or glycinergic [20, 21]. When sensory afferents are eliminated upon expression of diphtheria toxin [21], the GABAergic neurons that normally contact the sensory afferents still elaborate processes near motor neurons but do not contact the motor neurons (see Fig. 2b). These GABAergic inhibitory terminals subsequently retract. The molecular basis of this specificity in inhibitory synaptic partner matching relies on the expression of the immunoglobulin (Ig) superfamily protein, NB2 (Contactin 5) and the contactin-associated protein Caspr4, by the sensory afferents, and the expression of two L1 Ig family proteins, CHL1 and NrCAM, on the GABAergic interneurons providing inhibition onto the sensory afferents terminals [22]. Thus, inhibitory connections within the spinal cord are highly specific between each interneuron type and their postsynaptic partner.

In some circuits, inhibitory neurons adopt additional mechanisms that allow them to connect with other inhibitory neurons of the same type, without synapsing onto themselves (self-synapses or autapses). Cues thus exist to facilitate discrimination between ‘self’ and ‘non-self’ neurites. A key example of this common feature lies in the mammalian retina. γ-Protocadherins (Pcdhg), a family of adhesion molecules, permit GABAergic starburst amacrine cells (SAC) to synapse with neighboring SACs, without forming autapses [23]. Each SAC stochastically expresses one of 22 variants of Pcdhg, allowing the neurites of an individual cell to repel each other via homophilic repulsion, a process called ‘self-avoidance’ [23]. Conditional knockdown of all Pcdhg isoforms in the retina prevents SAC neurite self-avoidance, causing the neurites of an individual SAC to clump together and form autapses [24]. Expression of only one Pcdhg isoform in all SACs restores SAC self-avoidance, but also causes a reduction in neurite overlap between different SACs [23]. Electrophysiological recordings from pairs of SACs in retinas in which all SACs express the same Pcdhg, revealed reduced number and strength of inhibitory synapses between SACs [24]. *Pcdhg* genes have also been found to regulate the self-avoidance of cerebellar GABAergic Purkinje cell dendrites in a similar manner to SACs [23]. Pcdhgs thus play a central role in maintaining self-avoidance of neurites of inhibitory neurons in different CNS circuits.

In summary, inhibitory neurons use molecular cell-cell recognition cues to co-stratify with synaptic partners, to form specific synaptic partnerships, and to prevent the formation of autapses.

## Specific patterns of wiring amongst chosen partners

**Fig. 3:**
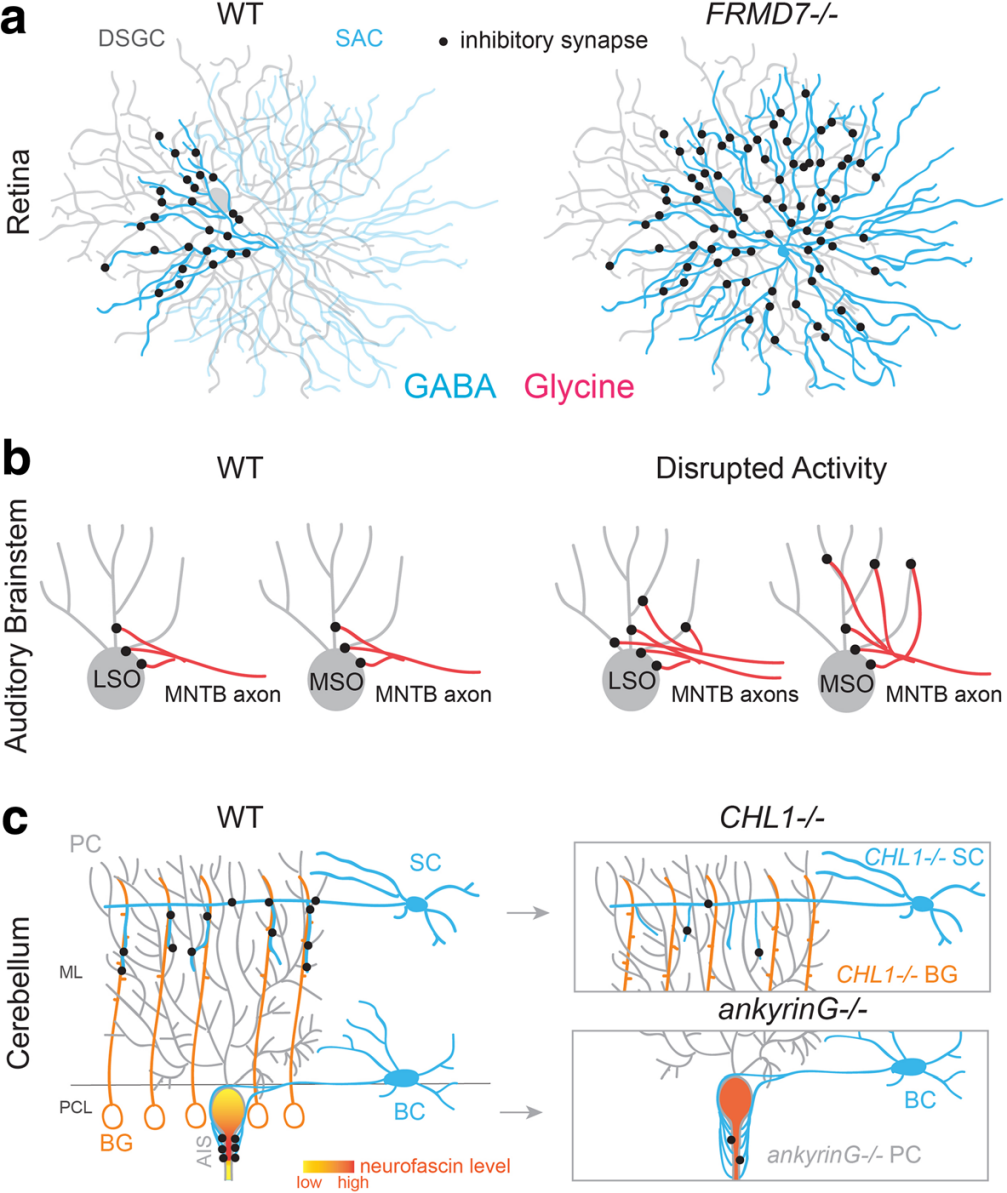
Mechanisms that regulate pre- and postsynaptic subcellular targeting of inhibitory connections. **a** In wildtype (WT) mouse retina, only a specific quadrant of the arbor of GABAergic starburst amacrine cells (SACs) form inhibitory synapses onto direction-selective retinal ganglion cells (DSGCs). In *FRMD7−/−* mice, this pattern of connectivity between SACs and DSGCs that prefer horizontal movement is disrupted. Summarized from [25]. **b** During normal development, excess MNTB axon targeting individual LSO neurons are eliminated. In the gerbil auditory brainstem, MNTB neurons initially provide inhibition to MSO neurons across their soma and dendritic arbor, but during development, dendritic synapses are eliminated after the onset of binaural input. Disrupted activity, such as loss of glutamate release or disrupted binaural input, prevents synapse elimination during development. Summarized from: [28, 117, 134–137]. **c** In the cerebellum, GABAergic stellate cells (SC) and basket cells (BC) utilize distinct cellular mechanisms to target distal dendrites and axon initial segments (AIS) of Purkinje cells (PC). In WT mice, ankyrinG binds to neurofascin and both are highly expressed in the AISs of PCs. Accordingly, in ankyrinG−/− mice the expression pattern of neurofascin is disrupted and basket cell processes erroneously target PC soma and distal processes, following the perturbed neurofascin expression pattern. The number of inhibitory synapses from basket cell to PC AISs is also reduced. In wildtype mice, stellate cells follow processes of Bergmann glia (BG) to make contact with distal dendrites of PCs. Both SCs and BGs express the cell surface molecule (CHL1). Consequently, in CHL1−/− mice stellate cells cannot recognize processes of BG and the number of SC synapses onto PC distal dendrites is reduced. Summarized from [33, 36]. ML: Molecular layer; PCL: Purkinje cell layer

Even after appropriate partners are selected, mechanisms are needed to establish stereotypic patterns of connectivity between inhibitory neurons and their postsynaptic partners. A remarkably selective pattern of connectivity occurs between the SACs and direction-selective ganglion cells (DSGCs), a circuit that is responsible for generating direction-selective output from the retina. Each quadrant of the arbor of a SAC preferentially forms synapses with one of four types of DSGCs that respond to motion in one of the four cardinal directions (dorsal, ventral, temporal and nasal) [25]. In the absence of the gene *FRMD7*, which encodes a member of the FERM domain of proteins that is enriched in SACs [25, 26], ‘horizontally tuned’ DSGCs receive erroneous connections with other quadrants of the SAC arbor, resulting in a loss of directional tuning in these cells [25] (see Fig. 3a).

A common feature of inhibitory circuits in the hippocampus, cerebellum, and cortex is the targeting of inhibitory synapses onto specific subcellular compartments of the postsynaptic cell. In the visual cortex, experience-independent mechanisms regulate the subcellular specificity of GABAergic innervation from distinct inhibitory interneurons onto glutamatergic pyramidal cells [27] (see also Fig. 1c). For example, both basket cell interneurons and bitufted cell interneurons accurately target pyramidal cell somas and distal dendrites, respectively, even when cortical tissue is removed at an early developmental stage and cultured in vitro for several weeks [27]. These results reveal that subcellular specificity can be achieved even in the absence of normal activity in the circuit and could likely be mediated by molecular cues. In contrast, activity-dependent mechanisms underlie the subcellular specificity of glycinergic innervation onto excitatory neurons of the gerbil medial superior olive (MSO) [28]. In the adult, the MSO neurons receive glycinergic inputs mainly at their cell body and proximal dendrites. However, glycinergic synapses are initially also present on the distal dendrites of MSO neurons, and these synapses are eliminated only after the onset of normal binaural hearing [28] (see also Figs. 1d and 3b) (reviewed in [29], see also Circuit refinement and maintenance).

The molecular mechanisms underlying subcellular targeting between inhibitory cell types are better understood compared to mechanisms regulating subcellular targeting of inhibitory neurons onto excitatory cells. For example, mechanisms underlying patterning of connections between GABAergic interneurons have been extensively studied in the cerebellum (reviewed in [30]). GABAergic basket cells and stellate cells use different cell adhesion molecules to target the AIS and distal dendrites of GABAergic Purkinje cells, respectively. Purkinje cells secrete Sema3A, which induces the expression of the semaphorin receptor neuropilin-1 (NRP1) in basket cell axons promoting the branching of basket cell axons specifically in the Purkinje cell layer [31, 32]. NRP1 then binds neurofascin, a member of the L1 family of immunoglobulin cell adhesion molecules, expressed by the Purkinje cell [32]. Basket cell processes expressing NRP1 follow the neurofascin gradient on the Purkinje cell away from the soma to eventually land on the AIS [33] (Fig. 3c). The neurofascin gradient is formed when ankyrinG, a membrane-skeletal protein expressed within the Purkinje cell, binds neurofascin and restricts its subcellular localization to the axon initial segment [33–35]. Accordingly, deletion of ankyrinG from Purkinje cells disrupts the neurofascin gradient and causes a dramatic reduction in the number of basket cell synapses on the Purkinje cell AIS [33] (Fig. 3c). In contrast, stellate cells target the distal dendrites of Purkinje cells through the guidance of Bergmann glia, mediated by expression of the cell surface molecule, CHL1, in both the Bergmann glia cells and the stellate cells [36] (summarized in Fig. 3c). These studies reveal that distinct molecular cues can direct the subcellular specificity of GABAergic connections even onto a single GABAergic postsynaptic partner.

By comparing the mechanisms underlying the subcellular specificity of inhibitory connections onto excitatory and inhibitory postsynaptic partners, it is evident that both activity-dependent and independent mechanisms can be utilized in a circuit-specific manner. Compared to GABAergic connections, much less is known about the molecular mechanisms that direct the subcellular targeting of synapses both onto glycinergic interneurons and onto the postsynaptic partners of glycinergic interneurons. For example, GABAergic DACs form a ring of synapses around the cell body of glycinergic AII amacrine cells of the retina [37]. The underlying mechanisms directing the specificity of this connection remain as yet unknown.

## Inhibitory synapse assembly

Synapse formation requires the coordinated accumulation of transmitter release machinery at presynaptic sites and clustering of appropriate receptors at postsynaptic locations. Studies across brain regions have shown that transmitter release is not essential for excitatory or inhibitory synaptogenesis. Complete blockade of glutamate and GABA release [38], blocking GABAergic transmission specifically [39–42], eliminating glycine transporter function [43, 44] or blocking vesicular release of both GABA and glycine [45] does not prevent synapse formation. Much work in the past and in recent years has thus focused on uncovering the complex molecular interactions that regulate precise pre- and postsynaptic assembly. The functional properties of GABA and glycine receptors are defined in part by their receptor subunit composition, which determines postsynaptic response kinetics [46]. Receptor composition varies within and across brain regions, and even across cell compartments of an individual neuron. Both GABA and glycine receptors are heteropentameric ligand-gated chloride channels (reviewed in [47]) with great diversity in subunit composition. Whereas most glycine receptors are composed of α-subunits (1-4) together with a single β-subunit type [48], most native GABA_A_ receptors in the brain display a two α(1-6), two β(1-4), and one γ subunit stoichiometry [49]. A GABA_A_ receptor composed of α1, β2, and γ2 in a 2:2:1 ratio is the most common native receptor type [50, 51]. When considering the organization of an inhibitory synapse, it is important to identify the GABA or glycine receptor composition opposite the presynaptic terminal because, as discussed below, receptor subtype dictates distinct protein interactions with synapse organizing molecules, scaffolding proteins and intracellular signaling molecules.

Outlined below are key molecular players currently known to have important roles in the formation of GABAergic and glycinergic synapses of the CNS (for a complete list of inhibitory synapse proteins see review [52]). We will compare the developmental steps and synapse organizing proteins for GABAergic versus glycinergic circuits wherever possible.

### Transsynaptic organizing proteins

During synapse formation, transsynaptic binding proteins bring pre- and postsynaptic membranes in close juxtaposition to ‘build’ a synapse. Transsynaptic proteins can also promote synaptic differentiation, organize postsynaptic scaffolding and signaling proteins, and play a role in the maintenance of the synapse (for review see [52, 53]). One well-characterized transsynaptic interaction is that of presynaptic proteins Neurexins (Nxns) with postsynaptic binding partners, Neuroligins (NLs) [53, 54]. Presynaptic Nxns can bind diverse postsynaptic partners: αNxns can bind to NL2, Calsyntenin-3 or dystroglycan, and βNxn can bind to NL1-3 isoforms depending upon the Nxn splice sites [55–57]. Individual postsynaptic organizers can also bind multiple presynaptic partners. For example, NL2 can bind either βNxn1 to promote synapse formation or MDGA1 (MAM domain-containing glycosylphosphatidylinositol anchor), which prevents NL2-Nxn binding and thus suppresses synapse formation [58, 59]. Interactions of transsynaptic organizers can also be isoform specific: MDGA1 only binds to NL2 and no other NL isoforms, and Calsyntenin-3 specifically binds to αNxn but not βNxn isoforms [55, 58, 60].

Many transsynaptic protein families are found at both excitatory and inhibitory synapses; however, specific isoforms are typically found at either excitatory or inhibitory synapses. For example, postsynaptic Slitrk1 and 2 bind to presynaptic protein tyrosine phosphatase (PTP)σ to promote excitatory synapse formation whereas Slitrk3 binds PTPδ to induce inhibitory synapse formation [61, 62]. Similarly, whereas αNxn and NL2 isoforms are expressed at inhibitory synapses, βNxn and NL1 are predominantly found at excitatory synapses [63–67]. Thus, distinct transsynaptic protein isoforms organize the establishment of excitatory versus inhibitory synapses.

Amongst inhibitory synapses, distinct NL isoforms guide the maturation of GABAergic and/or glycinergic synapses. In the retina, NL2 and NL3 are found predominantly at GABAergic synapses, whereas NL4 localizes preferentially at glycinergic synapses [68–70]. NL4 also colocalizes with glycine receptors in spinal cord and brainstem [70]. Furthermore, loss of NL2 or NL3 leads to the loss of structurally and functionally distinct subsets of GABA_A_ receptors in the retina: NL2 loss causes downregulation of the number of GABA_A_α3 and GABA_A_γ2-containing receptors in the inner synaptic layer of the retina [69], whereas NL3 loss causes reduction of GABA_A_α2-containing receptors [68]. Deletion of NL4 on the other hand is correlated with a loss of GlyRα1-containing retinal glycine receptors [70]. In the hippocampus of the NL4 knockout mouse, however, there is a loss of GABA_A_γ2-containing perisomatic synapses within the CA3 region of the hippocampus [71]. Lastly, deleting NL1-3 leads to a decrease in GABA_A_, but not glycine receptor clustering within the respiratory brainstem center [72]. Taken together, distinct transsynaptic protein isoforms contribute to the formation of varied subsets of GABAergic or glycinergic synapses in a brain region-specific manner.

Observations from NL deletion mutants suggest that at least one transsynaptic binding protein family can act at both GABAergic and glycinergic synapses. However, further studies are needed to determine whether other known protein families serve a similar role or whether some proteins are uniquely responsible for organizing glycinergic compared to GABAergic synapses. Different splice variants of Nxns and NLs can be directed to GABAergic or glutamatergic synapses [59, 73, 74], but it is not known if distinct splice variants of transsynaptic proteins are directed similarly to GABAergic versus glycinergic synapses. A combination of the transsynaptic protein splice variants expressed and the availability and regulation of intra- and extracellular binding partners can all contribute towards determining the type of inhibitory synapse that is assembled.

### Postsynaptic scaffolding proteins

Neurotransmitter receptors are recruited and stabilized at the inhibitory postsynapse by scaffolding proteins [52, 75]. Both GABA and glycine receptors can bind to gephyrin, a key inhibitory postsynaptic scaffolding protein. However, gephyrin plays distinct roles at glycinergic synapses compared to GABAergic synapses. Whereas all glycine receptors bind gephyrin, only a subset of GABA_A_ receptors show direct interactions with gephyrin. Specifically, gephyrin binds to the β-subunits of the glycine receptor [76], and there is only one gene encoding the glycine receptor β-subunit, which is expressed almost ubiquitously at all glycinergic synapses [48]. On the other hand, gephyrin binds α-subunits of the GABA_A_ receptor, specifically α1, 2, 3, and 5 [77–81]. Each isoform of the GABA_A_ α-subunit is expressed at a subset of GABAergic synapses, and multiple α-subunits can be present within a single GABA receptor at some synapses [51, 82]. Gephyrin binds glycine and GABA receptors at overlapping binding sites, which leads to mutually exclusive binding of GABA or glycine receptor subunits [83]. Moreover, there are differences in the affinity with which gephyrin binds glycine versus GABA receptor subunits (GlyR-β binding affinity > > GABA_A_-α binding affinity) [83], revealing that receptor type and receptor availability can influence how a scaffolding protein organizes receptor clustering at a specific postsynapse. Even between GABA_A_ α-subunits there are differences in gephyrin binding affinity. When the GABA_A_α5 gephyrin-binding site is replaced with the homologous sequence from GABA_A_α2, more receptors cluster at synapses indicating that the α2 subunit binds gephyrin at a higher affinity compared to GABA_A_α5 [81]. Gephyrin binding can also be a dynamic process: GABA_A_α5 binding with gephyrin shifts the balance of GABA_A_α5 receptor clustering to synaptic sites instead of extrasynaptic locations [81]. Binding to gephyrin, therefore, allows differential recruitment of GABA_A_ receptor subsets to synaptic versus extrasynaptic sites, and the amount of gephyrin recruited to the postsynaptic membrane controls receptor content, strength and sensitivity of the inhibitory synapse [84]. The differences in the affinity with which gephyrin binds glycine receptors versus different GABA receptor subtypes allows specific control of receptor expression and clustering within an individual inhibitory postsynapse.

It should be noted, however, that unlike glycinergic synapses gephyrin is not expressed at all GABAergic synapses. For example, in the retina, gephyrin colocalizes predominantly with GABA_A_ receptors containing the γ2, α2 and α3 subunits [85]. Accordingly, GABA_A_ receptors containing the γ2, α2, and α3 subunits are significantly reduced in the retina of gephyrin null mutant animals [86]. In the spinal cord and hippocampus, gephyrin knockdown similarly affects GABA_A_ receptors with γ2, α2, and α3 subunits [87, 88]. Taken together these results suggest that gephyrin is required for the proper synaptic trafficking of all glycine receptors but only a subset of GABA_A_ receptors ([86, 87, 89–92], recently reviewed in [93]). The complexity of these interactions introduces a rich diversity in how gephyrin regulates GABA versus glycine receptor clusters at inhibitory postsynapses of the CNS.

### Intracellular signaling molecules

Beyond scaffolding proteins, there are many intracellular signaling proteins involved in organizing the assembly of an inhibitory postsynapse. These proteins can be common to both GABAergic and glycinergic synapses. For example, collybistin, an inhibitory postsynaptic signaling protein, binds gephyrin, NL2, and NL4, and colocalizes at both GABAergic and glycinergic synapses throughout the CNS [70, 94–96]. Despite being present at both GABAergic and glycinergic synapses, collybistin is only required for recruiting intracellular gephyrin to a subset of GABAergic postsynapses and is not required for glycine receptor localization [97]. The ability of collybistin to cluster gephyrin depends on its conformational state, and collybistin activation requires proteins such as NL2 [95, 98], NL4 [70], or GABA_A_α2 [79]. A favored model for GABAergic postsynaptic differentiation relies on a tripartite NL2-collybistin-gephyrin complex, with NL2 stabilizing the active conformation of collybistin thereby allowing collybistin to localize to the membrane and create nucleation sites for gephyrin deposition and subsequent inhibitory receptor clustering [95, 98, 99]. In comparison, glycinergic synapses in some brain regions seem to rely on a NL4-dependent, but collybistin-independent mechanism of receptor clustering. More work is needed to resolve how NL4, gephyrin and glycine receptors are recruited at these glycinergic postsynapses.

In summary, gephyrin and collybistin are both present but play distinct roles at GABAergic and glycinergic postsynapses. Whereas gephyrin regulates receptor clustering at both GABAergic and glycinergic synapses, collybistin only regulates receptor clustering at GABAergic synapses. Additionally, much like gephyrin, collybistin specifically binds the GABA_A_α2 subunit over other GABA_A_ α-subunits [79], allowing for differential regulation of GABAergic synapses with distinct receptor subunit composition.

Recent technological advances in in vivo chemico-genetic and proteomic approaches have begun to identify novel inhibitory synapse organizing proteins and new roles for known synapse organizing proteins [100, 101]. These techniques tag known postsynaptic proteins with enzymes, which promote biotinylation within a small radius, allowing for the capture of other proteins at that synapse. Thus far gephyrin has been used to capture inhibitory synaptic proteins, leading to the discovery of novel synapse proteins such as Insyn1, which regulates GABA-mediated, but not AMPA-mediated currents in hippocampal neurons [100]. As gephyrin is expressed at both GABAergic and glycinergic synapses, it will be important to use GABAergic and glycinergic synapse-specific proteins with this approach in the future to identify and compare the array of proteins expressed at GABAergic versus glycinergic synapses. Additionally, in light of the differences in gephyrin and collybistin function for inhibitory synapse assembly despite overlapping expression patterns, it will also be important to determine whether there are conserved roles for synapse organizing proteins across inhibitory synapse types.

## Maturation of inhibitory circuits

**Fig. 4:**
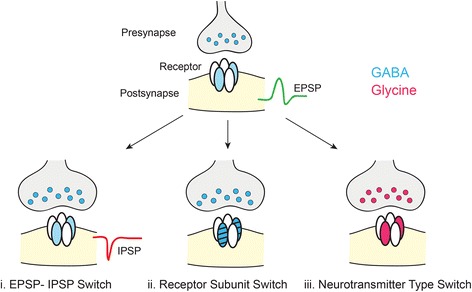
Maturational ‘switches’ at inhibitory synapses. i) GABAergic and glycinergic transmission is initially depolarizing early in development due to the high intracellular chloride concentration within the postsynaptic cell. Reversal of the chloride gradient with maturation leads to hyperpolarization upon activation of GABA and glycine receptors [104]. EPSP: excitatory postsynaptic potential, IPSP: inhibitory postsynaptic potential. ii) During maturation, the composition of GABA and glycine receptor pentamers switches to incorporate different subunits, typically resulting in faster synaptic transmission [122, 123]. iii) Inhibitory circuits can also undergo a neurotransmitter type switch accompanied by a change in postsynaptic receptor expression. The transition from GABA-releasing to glycine-releasing is more common, but the reverse has also been documented [112, 114], see text for more details

After synapse formation, cellular and molecular processes are engaged to promote the maturation of nascent connections. Inhibitory circuit maturation includes concomitant changes in the pre- and the postsynapse together with alterations in cellular properties such as the chloride gradient (summarized in Fig. 4). Presynaptic maturation of inhibitory neurons includes changes in transmitter type and release properties, and postsynaptic changes include alterations in receptor subunit composition.

### Cellular change in chloride gradient

During early circuit assembly, the intracellular chloride concentration of developing neurons is elevated compared to that of mature neurons [102]. Therefore, the chloride currents evoked upon GABA and glycine receptor activation is depolarizing at this stage [102, 103]. Developmental increase in the expression or activity of the chloride transporter KCC2 has been shown to reverse the chloride gradient within neurons leading to a developmental ‘switch’ in GABAergic and glycinergic transmission from depolarizing to hyperpolarizing [104, 105]. In the mouse CNS, this switch usually occurs at the end of the first postnatal week [103]. The activity of both GABA and glycine is thus largely depolarizing during initial synapse formation and circuit assembly, and GABA and glycine receptor-mediated signals switch from depolarizing to hyperpolarizing via a shared KCC2 mechanism. Activation of GABA_A_ receptors in the hippocampus and glycine receptors in the spinal cord are necessary for the increased expression and activity of KCC2, respectively [106, 107] indicating a role for both receptor types in mediating this switch.

### Changes in neurotransmitter type and release properties

During maturation, some inhibitory neurons switch from utilizing one neurotransmitter type to another. In the mammalian auditory brainstem nuclei and spinal cord, presynaptic interneurons in some circuits transition from releasing primarily GABA, or GABA and glycine, to predominantly releasing glycine [108–111]. In these brain regions, individual axon terminals have been found to undergo this change, although a small amount of GABA release is maintained at some mature synapses [109, 112]. In auditory brainstem circuits, the neurotransmitter switch is the result of both pre- and postsynaptic changes: greater number of presynaptic vesicles releasing glycine, with an increased glycine concentration per vesicle, together with increased glycine receptors and fewer GABA_A_ receptors at the postsynapse [109, 110, 112, 113]. Inhibition in some circuits can also transition from glycinergic to GABAergic. In the brainstem, the dorsal motor nucleus of the vagus (DMV) switches from mixed GABA-glycine to largely GABAergic during postnatal development [114]. In this brain region, a loss of glycinergic input, but not postsynaptic glycine receptors underlies the emergence of a predominantly GABAergic circuit [114]. Inhibition in some circuits within the nucleus tractus solitarius (NTS), a brainstem nucleus that projects to the DMV, transition from pure GABAergic to mixed GABA-glycine [115]. In the NTS, the developmental alterations are also presynaptic: most axon terminals are GABAergic at birth, but a subset of terminals within the lateral region of the NTS subsequently co-release GABA and glycine, and continue to do so at adulthood, although all regions of the NTS express both GABA_A_ and glycine receptors from birth [115]. In sum, both pre- and postsynaptic alterations can underlie a developmental switch in the type of inhibition (GABA or glycine), and the nature of the transition from one transmitter type to another seems to be circuit-specific. It would be interesting to determine whether change in neurotransmitter type during circuit maturation, as observed in some brain regions that co-release GABA and glycine, is a feature specific to these regions, or whether brain regions with inhibition provided by only GABAergic interneurons, such as the cortex, also undergo a similar change in neurotransmitter type.

In addition to changes in the type of neurotransmitter released, other features of inhibitory circuit maturation include an increase in quantal size and in synchronous neurotransmitter release [110, 116]. For example, an inhibitory projection from the medial nucleus of the trapezoid body (MNTB) to the lateral superior olive (LSO) undergoes a 12-fold increase in the strength of the postsynaptic current within the first two postnatal weeks [117], even though the number of connections from the MNTB to LSO decreases with circuit maturation (Fig. 3b). In the MNTB and ventral cochlear nucleus, glycine release becomes more synchronized as the inhibitory circuits onto MNTB and cochlear nuclei mature [110, 112]. The change in neurotransmitter release properties is likely regulated by changes in the presynaptic expression of calcium channels that mediate vesicle fusion. For example, in projections from the MNTB to the LSO, neurotransmitter release is initially mediated by L- and N-type calcium channels, but after hearing onset release is mediated by P/Q-type calcium channels, which have been shown to support synchronous release in some circuits [116, 118, 119].

### Changes in receptor expression

Postsynaptic receptor kinetics are determined in large part by receptor subunit composition [46, 120]. Across neural circuits, both GABA and glycine receptors undergo characteristic changes in receptor subunit expression during development. In particular, both receptor types initially contain subunits that confer slower response kinetics but at maturity incorporate subunits that mediate faster response kinetics [46, 121]. For example, initially glycine receptors typically contain the α2 subunit, but after circuit maturation contain the α1 subunit [122]. GABA receptors can also undergo a change from α2- to α1-containing, as well as from α5- to α3-containing configurations [121, 123, 124]. For glycine receptors, changes in inhibitory subunit expression during development appear to coincide with the transition of the action of glycine from depolarizing to hyperpolarizing. In cultured spinal cord neurons, loss of KCC2 activity impedes the switch of glycine receptors from α2 to α1-containing [125]. In KCC2 knockdown neurons, glycine receptor clusters containing the α1 subunit are fewer, smaller, and colocalize with less gephyrin compared to α2-containing clusters, but GABAergic transmission appears unchanged [125]. Therefore, hyperpolarizing inhibitory synaptic transmission is necessary for glycine but not GABA_A_ receptor maturation in the spinal cord. Receptor subunit changes during circuit maturation also speed response kinetics of excitatory synapses (see review [126]), so this switch during circuit maturation occurs across all neurotransmitter types. Faster postsynaptic inhibitory response kinetics due to receptor subunit switches can have functional consequences because the temporal properties of synaptic inhibition can shape signal integration, feature selectivity, and coincidence detection (reviewed by [127, 128]).

During maturation, synapses continue to accrue receptors to form larger postsynaptic clusters. Although inhibitory neurotransmission is not required for synapse formation, it is important for proper receptor clustering. Perturbing presynaptic release of either GABA- or glycine can lead to receptor accumulation deficits, but in a region-specific and receptor type-specific manner. For example, in the retina, when presynaptic GABA release is blocked or all inhibitory neurotransmission disrupted by loss of VIAAT, the vesicular inhibitory amino acid transporter, glycinergic synapses are unaffected, but many GABAergic synapses on bipolar cells fail to accumulate mature levels of receptors [40, 129]. Specifically, in the VIAAT knockout GABA_A_α1γ2-containing receptors on bipolar cell axons are downregulated whereas GABA_A_α1 receptors on bipolar cell dendrites are upregulated. Furthermore, loss of presynaptic GABAergic transmission in the retina causes a downregulation in GABA_A_α1, but not GABA_A_α3 or GABA_C_ρ-containing receptors within bipolar cell axon terminals [40]. Therefore, activity-induced changes in receptor expression can occur in a receptor-type specific, receptor subunit-specific, and a cell-compartment specific manner even within an individual neuron.

Interestingly, whereas loss of inhibitory neurotransmission does not alter glycine receptor expression in the retina [129], changes in activity do disrupt glycine receptor maturation in the spinal cord. In spinal cord cultures, using glycine receptor antagonists or L-type calcium channel blockers prevents glycine receptor clustering, but does not alter aggregation of synaptic GABA_A_ receptors [130]. Together, these observations suggest that there may be distinct mechanisms by which activity regulates the maturation of GABAergic versus glycinergic synapses in different regions of the CNS.

The level of synaptic transmission may also be important in regulating synapse maturation. For example, in the cortex, when presynaptic GABA release is suppressed within an individual basket cell interneuron, fewer and smaller inhibitory synapses are formed onto pyramidal neurons [131]. On the other hand, when GABAergic transmission from basket cells is abolished, more inhibitory synapses are made onto pyramidal neurons due to a deficit in synaptic pruning [41]. The differences in these manipulations suggest that inhibitory neurotransmission is important for synaptic competition and subsequent synapse maturation, but the synaptic consequences of blocking transmission depends on the extent of blockade.

Finally, GABA and glycine receptor aggregation at the postsynapse can be differentially regulated by activity-independent mechanisms such as microglial signaling. Cantaut-Belarif et al. [132] found that microglia specifically regulate the accumulation of glycine but not GABA_A_ receptors in spinal cord cultures. These authors found that microglia regulate the lateral diffusion of glycine receptors at synapses by releasing prostaglandin E2, which binds to neuronal EP2 receptors, shown to influence glycine receptor signaling [132, 133].

Comparing across circuits, there are shared mechanisms (e.g. chloride concentration changes) and common themes (e.g. faster neurotransmission) that emerge during the maturation of GABAergic and glycinergic circuits. However, inhibitory circuits can also be regulated differentially by both activity-dependent and activity-independent mechanisms, in a region-specific manner. Therefore, some aspects of inhibitory circuit maturation are highly specific to the circuit in question, revealing the importance of inquiry at individual circuits and synapses.

## Circuit refinement and maintenance

Both the distribution and number of inhibitory synapses onto postsynaptic targets determine how information is processed within a circuit. To achieve proper connectivity, circuits often undergo synapse elimination of inappropriate contacts and strengthening of preferred synaptic connections. Together, these two developmental processes lead to the establishment of correct wiring patterns, which are thereafter maintained.

### Circuit refinement

In both GABAergic and glycinergic circuits, more synapses are formed than will persist at maturity, thus requiring synapse elimination to establish the final connectivity patterns [28, 41]. Neural activity plays an important role in this refinement process. This is exemplified in the mammalian auditory system, which accurately determines the source of a sound by computing both the interaural time difference, the time delay between when a sound is heard in one ear versus the other, and the interaural level difference, the difference in sound intensity between the two ears (see Figs. 1d and 3b). To compute the interaural level and time differences, inhibitory connections from the MNTB must provide tonotopically-organized inhibition onto neurons in the LSO and temporally precise inhibition onto neurons in the MSO, respectively. Inhibitory connections from the MNTB to the LSO and MSO undergo extensive synaptic refinement during circuit maturation. Initially LSO neurons receive weak mixed GABA-glycine inputs from many MNTB neurons. However, after refinement, LSO neurons receive strong glycinergic input from a few MNTB neurons resulting in a more precise tonotopic organization [117] (see also Fig. 3b). This refinement requires excitatory neurotransmission [134–136], the correct pattern of spontaneous activity during development [137], and occurs before normal onset of hearing [117]. MNTB neurons co-release GABA, glycine, and glutamate during a brief window during development [134]. Case et al. [136] confirmed that the function of this glutamate release is to act as an excitatory neurotransmitter rather than facilitating GABA-glycine co-release. When this transient period of glutamate release is prevented by deletion of the glutamate transporter expressed within MNTB neurons, MNTB neurons fail to eliminate excess synapses, and the response amplitudes of MNTB connections do not increase as much as in wildtype animals [135]. Additionally, the pattern of spontaneous activity is also critical for proper MNTB-LSO circuit refinement. Clause et al. [137] found that disrupting the pattern, but not level of spontaneous activity was sufficient to prevent synapse elimination, synapse strengthening, and axonal pruning (see also Fig. 3b). Of note, functional loss of synapses in this circuit precedes axon terminal pruning by many days [117].

Release of inhibitory transmitters may also play a role in inhibitory circuit refinement. For example, in the MSO of gerbils, MNTB neurons initially provide glycinergic synapses onto the soma and dendrites of MSO neurons. The dendritic synapses onto MSO neurons are eliminated during development shortly after the onset of hearing (see Fig. 3b) [28], at which time glycinergic transmission is hyperpolarizing (reviewed in [138]). Disrupting the binaural input to the MSO by unilateral cochlear ablation or exposure to omnidirectional noise prevents synapse elimination of dendritic contacts and the corresponding refinement of MNTB axon terminal branches [28, 139] (see also Fig. 3b). Interestingly, within the MNTB-LSO circuit, hyperpolarizing activity mediated via GABA or glycine release is not required for circuit refinement [140]. This finding suggests that inhibitory circuits even within the same brain structure and using the same inhibitory neurotransmitters at maturity can rely on distinct signaling mechanisms (e.g. depolarizing versus hyperpolarizing) to regulate circuit refinement.

Within the cortex, inhibitory GABAergic transmission can shape synaptic connectivity patterns of interneurons. When GABAergic transmission is completely blocked from individual presynaptic basket cells of the visual cortex, the basket cell forms more, but smaller synapses onto the soma of pyramidal neurons both in vitro and in vivo even when transmission is blocked late in development [41]. Live-cell imaging revealed that basket cells form transient synapses onto pyramidal neurons; however, neurons lacking GABAergic transmission failed to eliminate many of these synapses [41]. Thus, GABAergic transmission appears necessary for activity-dependent competition and synapse refinement within a subpopulation of cortical interneurons.

Finally, structural refinement of the axonal arbor of an inhibitory neuron can occur without synaptic reorganization. In the cortex, inhibitory chandelier cells form stereotypical synapses onto the axon initial segment of excitatory pyramidal neurons (see Fig. 1c). Steinecke et al. [141] observed that during postnatal development, chandelier cell varicosities make functional synapses onto axon initial segments (on-target) of the pyramidal neurons but also have off-target varicosities. The off-target varicosities are, however, preferentially retracted as the chandelier cell matures and do not contain presynaptic markers [141]. Therefore, inhibitory neurons can target their synapses with subcellular specificity from the outset, but continue to refine their axonal branching patterns. Thus, synaptic and structural refinement can be regulated separately during development.

### Circuit maintenance

Once the proper pattern of synaptic connectivity is established, the circuitry must be maintained. Synapse-associated proteins involved in circuit development can also be necessary for circuit maintenance. For example, dystroglycan, a transsynaptic binding protein located at the postsynapse is important for both the formation and maintenance of CCK-positive basket cell contacts onto pyramidal neurons. When dystroglycan is specifically eliminated from pyramidal neurons in early development, axons of CCK-positive GABAergic neurons fail to innervate the pyramidal neurons; however, there is little change in the number of GABAergic synapses onto pyramidal neurons, suggesting that other presynaptic partners could increase synaptic contacts to compensate for the loss of CCK-positive contacts [142]. On the other hand, eliminating dystroglycan from pyramidal neurons in the adult mouse results in a progressive loss of CCK-positive basket cell innervation over time, revealing a continued role for dystroglycan in maintaining these inhibitory connections [142]. Together these observations reveal that: (i) transsynaptic binding proteins important for circuit development can also be necessary for circuit maintenance, and (ii) transsynaptic binding proteins can dictate connectivity of cellular partners during circuit assembly independent of synapse number.

Beyond maintaining synaptic partner contact, synaptic proteins can play a role in maintaining receptors at the inhibitory postsynapse. For example, as described in Inhibitory synapse assembly, collybistin is necessary for the synaptic localization of gephyrin at a subset of GABAergic synapses within the hippocampus, and the loss of collybistin during development prevents clustering of GABA_A_ receptors at dendritic synapses of hippocampal pyramidal neurons [97, 143]. Loss of collybistin after synapse formation also results in a gradual loss of both gephyrin and GABA_A_ receptor expression at dendritic pyramidal synapses [143], implying that collybistin is required for the continued maintenance of gephyrin and consequently GABA_A_ receptors at those hippocampal synapses.

Whereas dystroglycan and collybistin are necessary for the maintenance of subsets of GABAergic synapses, gephyrin dynamically regulates receptor clustering at both GABAergic and glycinergic synapses. Gephyrin-binding of glycine receptors not only increases the rate at which receptors reach the plasma membrane [144], but it also increases the time that receptors reside at the postsynapse [145]. Consequently, in spinal cord cultures, introducing truncated forms of gephyrin, which fail to properly trimerize, can displace glycine receptors from postsynapses and lead to glycine receptor endocytosis [145]. These observations reveal that glycine receptors are actively maintained in the plasma membrane and at the postsynapse by gephyrin binding. Gephyrin also stabilizes GABA_A_ receptors at the postsynapse. Knockdown of gephyrin in hippocampal cultures decreases the number and stability of GABA_A_ receptor clusters, but does not affect receptor membrane insertion [146]. Therefore, gephyrin not only organizes the formation of inhibitory synapses, but it also continues to dynamically regulate receptor clustering at inhibitory postsynapses. As the same synaptic molecules can be employed for both the formation and maintenance of CNS inhibitory circuits, understanding the mechanisms involved in synapse development can provide insight into the molecular pathways that also maintain synapses in the mature circuit.

## Cross-talk in the regulation of GABAergic and Glycinergic synapses

**Fig. 5:**
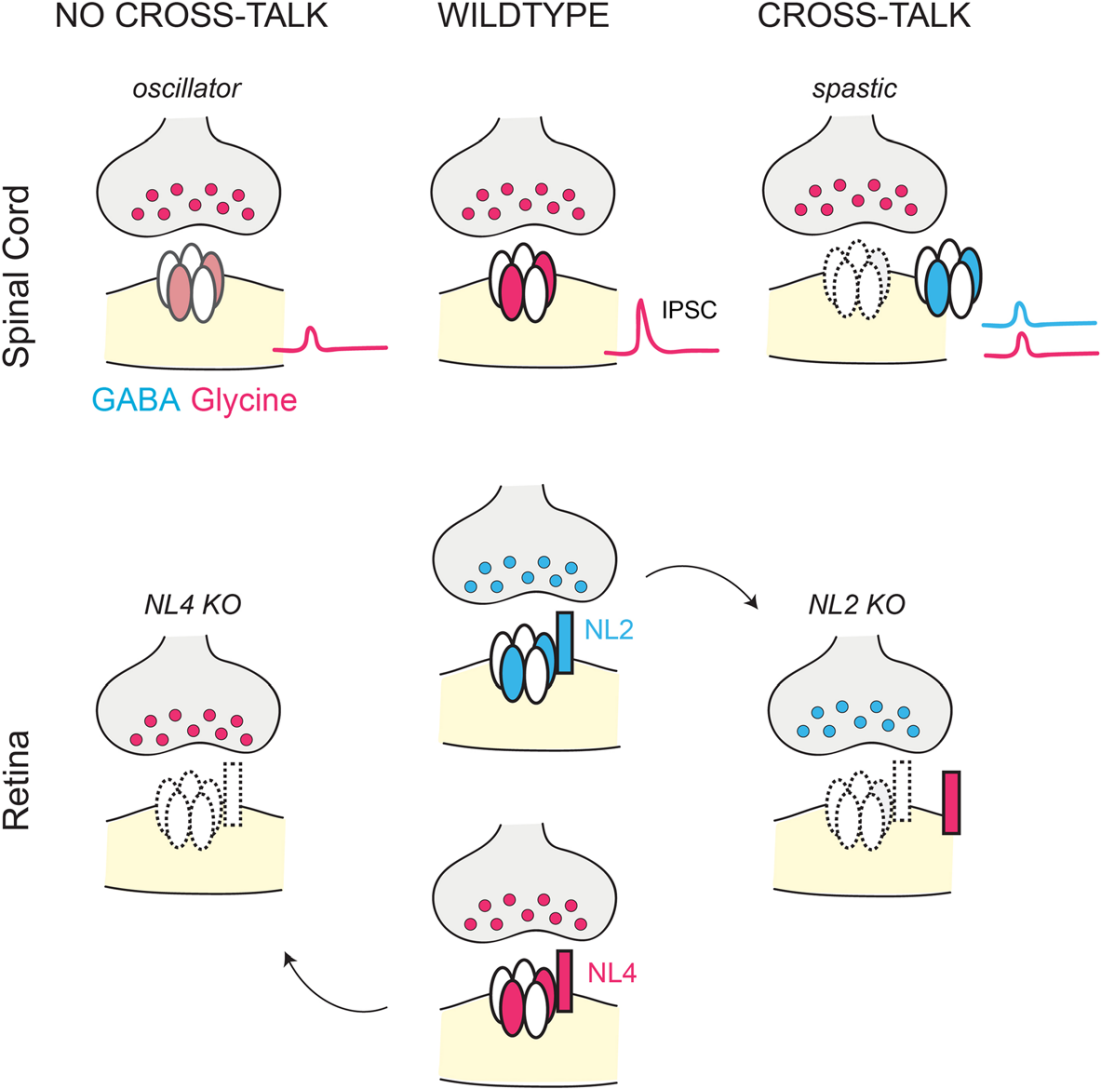
Cross-talk between inhibitory neurotransmitter circuits. In some circuits, perturbing either GABAergic or glycinergic signaling leads to potentially compensatory postsynaptic changes. In both the spinal cord and retina, there are conditions in which there is cross-talk between inhibitory neurotransmitter circuits. In the spinal cord, *oscillator* mice carry a mutation that results in non-functional glycine receptors (non-α1 subunit containing glycine receptors, faded) and *spastic* mice carry a mutation that results in a dramatic reduction of glycine receptors at the synapse (dotted lines). Both mutations result in decreased glycinergic inhibitory postsynaptic currents (IPSCs, red traces). However, in the *spastic* mice there is an increase in extrasynaptic GABA_A_ receptors and in the amplitude of GABAergic IPSCs (blue trace) [149, 150]. In the wildtype retina, Neuroligin 2 (NL2) is found at GABAergic synapses, and NL4 is localized at glycinergic synapses. In the retina of a NL4 knockout (KO) animal, α1-containing glycine receptors are lost, but there is no change in the expression of other NLs. However, in the NL2 KO retina, GABA_A_α3 and GABA_A_γ2-containing synapses are down-regulated, and there is an up-regulation of NL4 [69, 70]

In most circuits of the CNS, the development and function of GABAergic and glycinergic connections are independently regulated. For example, GABA_A_, GABA_C_ and glycine receptor synapses are all present on an individual retinal bipolar cell axon terminal, but loss of GABA_A_ receptors within these axon terminals does not cause alterations in the expression of neighboring GABA_C_ or glycine receptor clusters [40]. There are examples, however, of ‘cross-talk’ between the mechanisms regulating the development of GABAergic and glycinergic circuits when either circuit is perturbed (summarized in Fig. 5). During early development, loss of a transsynaptic binding protein typically associated with GABAergic synapses can lead to an increase in the expression of a transsynaptic binding protein associated with glycinergic synapses. GABA_A_ receptors in the retina colocalize with NL2 and those in hippocampus are dependent on NL4 expression [69, 71]. When NL2 is eliminated, GABA_A_α3-containing and GABA_A_γ2-containing synapses within the retina are lost. Under this condition, expression of NL4, which is typically observed at retinal glycinergic synapses, is upregulated in the retina [69, 70]. The reverse does not seem to hold true: when NL4 is eliminated and glycine α1-containing receptors are lost in the retina, expression of other NL isoforms remains unaffected in the retina [70]. However, in the hippocampus of NL4 knockout mice, when GABA_A_γ2-containing synapses are lost, there is an upregulation of NL2, which has been shown to influence both GABAergic and glycinergic transmission in the CNS and GABA_A_ receptor clustering in the hippocampus [71, 95, 147]. These findings suggest that the expression of distinct NL isoforms (specifically NL2 and NL4) can be regulated in a dependent manner (Fig. 5); however, compensatory changes in NL expression have thus far only been documented following the loss of GABA_A_ receptor - associated NL expression and not after the loss of glycine receptor-associated NL expression.

Changes in glycine receptor expression can also lead to alterations in GABAergic circuits. For example, *spastic* (*spa*) mice carry a mutation that causes a significant reduction in GlyRβ subunit expression and hence reduced numbers of synaptic glycine receptors and amplitude of glycinergic postsynaptic currents [148, 149]. Spinal cord neurons from these animals show larger amplitude of GABAergic postsynaptic currents compared to control mice [149] (Fig. 5). Therefore, decrease in inhibition mediated by one transmitter type can be accompanied by an increase in the transmission mediated by the other transmitter type i.e. GABAergic currents can replace glycinergic postsynaptic currents. The cellular mechanisms that underlie these potentially compensatory circuit alterations in the *spa* mice have not yet been determined and would be interesting to unravel in the future.

Not all mutants with reduced glycinergic currents show compensatory GABAergic circuit alterations. *Oscillator* (*ot*) mice carry a mutation that causes production of a non-functional GlyRα1 subunit, but receptors without the non-functional subunit remain at the synapse [149]. These alterations also result in reduced amplitude of glycinergic postsynaptic currents [148]. However, spinal cord neurons from *ot* animals do not show any alteration in GABAergic postsynaptic currents [149] (Fig. 5). Therefore, whereas spinal cord neurons from animals with either *spa* or *ot* mutations have reduced glycinergic inhibition, only *spa* mice show increased GABAergic signaling. The kinetics of GABAergic and glycinergic responses do not change in either of these mutants, suggesting that receptor subunit composition remains unchanged in both these mutants [149].

In addition to changes in postsynaptic currents, GABAergic and glycinergic axons have also been shown to undergo presynaptic changes following postsynaptic receptor alterations. Neurons in the hypoglossal nucleus of *spa* mice have decreased synaptic glycine receptor expression and increased extrasynaptic GABA_A_ receptor expression compared to wildtype animals [150]. In normal conditions, the presynaptic terminals onto the neurons of the hypoglossal nucleus are largely glycinergic or contain both GABA and glycine, but with maturation, more terminals become purely glycinergic [151]. In the *spa* mouse, however, presynaptic terminals onto neurons of the hypoglossal nucleus are largely GABAergic during development and remain so as the circuit matures [150]. These differences indicate that changes in postsynaptic receptor expression can induce a corresponding presynaptic change in neurotransmitter content. Together, these studies reveal that in some conditions when inhibitory neurotransmitter signaling is altered, inhibitory circuits have the capacity to modify the neurotransmitter type and postsynaptic response properties, suggesting that the mechanisms regulating GABAergic and glycinergic synapse formation do interact in some CNS circuits.

## Conclusions

Comparing the assembly, maturation and maintenance of GABAergic and glycinergic circuits, as well as circuits in which GABA and glycine are co-released, suggests three areas which need to be explored further:

(i) Both GABAergic and glycinergic neurons have been shown in some brain regions to innervate specific laminae within the neuropil, or target postsynaptic partners in a cell-compartment specific manner. Although the cellular and molecular mechanisms guiding partner selection and subcellular specificity of connections involving GABAergic interneurons are being unraveled, such mechanisms have yet to be determined for glycinergic interneurons.
(ii) GABAergic and glycinergic synapses are diverse in structure and function across the CNS. Individual circuits and/or synapses utilize specific mechanisms, both activity-dependent and independent, to control their formation, maturation and maintenance. Thus, studies that examine the effects of either removing a synapse organizing protein or altering network activity should be circuit or cell-type specific to enable an understanding of inhibitory circuit formation at the level of individual synapses. Moreover, to distinguish the mechanisms regulating synapse maturation and maintenance, comparisons need to be made with circuit alterations performed after synapse formation.
(iii) Extensive studies of the auditory brainstem nuclei of mammals have revealed a role for activity in the refinement and maturation of circuits in which GABA and glycine are co-released at a synapse. The proteins involved in synapse formation and maintenance of these types of synapses are less well understood. Mechanisms regulating the development and refinement of such connections found in brainstem, spinal cord, and recently, in the midbrain [152] also remain largely unknown. It would be particularly interesting to discover whether synapse organizing proteins that establish connections at which GABA and glycine are co-released differ from those that regulate purely GABAergic or purely glycinergic connections.

Comparing the development and maintenance of the various inhibitory circuit types could provide novel insights into the basis of circuit dysfunction following disruption in one or both inhibitory neurotransmitter types, and in doing so, offer therapeutic options for re-establishing normal function.

## Abbreviations

AIS: Axon initial segments
CNS: Central nervous system
DAC: Dopaminergic amacrine cell
DMV: Dorsal motor nucleus of the vagus
DSGC: Direction-selective ganglion cells
GABA: γ-aminobutyric acid
GlyR: Glycine receptor
Ig: Immunoglobulin
LGN: Lateral geniculate nucleus
LSO: Lateral superior olive
MDGA: MAM domain-containing glycosylphosphatidylinositol anchor
MNTB: Medial nucleus of the trapezoid body
MSO: Medial superior olive
NL: Neuroligin
NRP1: Neuropilin-1
NTS: Nucleus tractus solitarius
Nxn: Neurexin
ot: Oscillator
Pcdhg: γ-Protocadherins
PTP: Protein tyrosine phosphatase
SAC: Starburst amacrine cell
sdk: Sidekick
Sema: Semaphorin
spa: Spastic
VIAAT: Vesicular inhibitory amino acid transporter
